# Adjusting Heterodimeric Coiled-Coils (K/E Zipper) to Connect Autophagy-Inducing Peptide with Cell-Penetrating Peptide

**DOI:** 10.3390/pharmaceutics15041048

**Published:** 2023-03-24

**Authors:** Yoshiyuki Hakata, Kazuma Yamashita, Sonoko Hashimoto, Takashi Ohtsuki, Masaaki Miyazawa, Mizuki Kitamatsu

**Affiliations:** 1 Department of Immunology, Faculty of Medicine, Kindai University, 377-2 Ohno-Higashi, Osakasayama 589-8511, Japan; hakata@med.kindai.ac.jp (Y.H.);; 2 Department of Arts and Sciences, Faculty of Medicine, Kindai University, 377-2 Ohno-Higashi, Osakasayama 589-8511, Japan; 3 Department of Applied Chemistry, Faculty of Science and Engineering, Kindai University, 3-4-1 Kowakae, Higashiosaka 577-8502, Japan; 4 Department of Interdisciplinary Science and Engineering in Health Systems, Okayama University, 3-1-1 Tsushimanaka, Okayama 700-8530, Japan

**Keywords:** autophagy-inducing peptide, cell-penetrating peptide, leucine zipper, drug delivery system

## Abstract

A connection of a functional peptide with a cell-penetrating peptide (CPP) used a heterodimeric coiled-coil as a molecular zipper can improve the intracellular delivery and activity of the functional peptide. However, the chain length of the coiled coil required for functioning as the molecular zipper is unknown at present. To solve the problem, we prepared an autophagy-inducing peptide (AIP) that conjugates with the CPP via heterodimeric coiled-coils consisting of 1 to 4 repeating units (K/E zipper; AIP-Kn and En-CPP), and we investigated the optimum length of the K/E zipper for effective intracellular delivery and autophagy induction. Fluorescence spectroscopy showed that K/E zippers with n = 3 and 4 formed a stable 1:1 hybrid (AIP-K3/E3-CPP and AIP-K4/E4-CPP, respectively). Both AIP-K3 and AIP-K4 were successfully delivered into cells by the corresponding hybrid formation with K3-CPP and K4-CPP, respectively. Interestingly, autophagy was also induced by the K/E zippers with n = 3 and 4, more intensively by the former than by the latter. The peptides and K/E zippers used in this study did not show significant cytotoxicity. These results indicate that the effective induction of autophagy occurs via an exquisite balance of the association and dissociation of the K/E zipper in this system.

## 1. Introduction

The development of methods for the intracellular delivery of functional peptides and proteins will help control cellular activities and could promote the creation of novel functional peptides as pharmaceuticals. In such intracellular delivery, a method using cell-penetrating peptides (CPPs) as carriers [1,2,3,4,5,6] can be accomplished by directly conjugating the CPP to a functional peptide (cargo) that does not itself have the ability to enter cells. This method is convenient because it enables the cargo conjugated with a CPP to be spontaneously carried into cells. In addition, the function of cargo–CPP conjugates can easily be optimized on the basis of amino acid sequences, and these conjugates have unlimited potential as new molecular pharmaceuticals. In particular, peptide–CPPs as short as 50 mer can be readily synthesized by solid-phase peptide synthesis (SPPS).

Although the direct covalent attachment of a CPP to a functional peptide is a promising method, some problems still remain unresolved. One of these is the intracellular separation of CPP from its cargo. CPP must of course be linked to the cargo until the cargo–CPP is delivered into cells, but their dissociation after delivery inside cells is desirable. Because numerous CPPs have an abundance of positive charges in their sequence, conjugating them with cargo may cause nonspecific interactions with intrinsic proteins and/or negatively charged RNA and DNA within cells after delivery. In this context, there is concern that the bioactivity of the cargo would be suppressed [7,8,9].

As a simple molecular design to circumvent this problem, a strategy of separating functional peptides and CPPs after intracellular delivery is considered to be effective. Such molecular designs have been reported by several research groups [9,10,11,12], especially using disulfide bonds [13,14,15]. On the other hand, we have worked on the development of molecular zippers that connect CPP with cargo outside cells and separate them inside cells by using a noncovalently linked hybrid module [16,17,18,19].

Leucine zipper peptides (Lz) have been reported by Bosshard et al. [20,21], which include a peptide containing lysine (LzK) and a peptide containing glutamic acid (LzE) at appropriate positions. Since LzK and LzE form a specific 1:1 hybrid with a parallel orientation, we used them as a molecular zipper to link the functional peptide and CPP. We previously synthesized an autophagy-inducing peptide (AIP) derived from Beclin-1 protein [22], Nanog protein [23,24], GFP protein, or p53 protein [25] in the form of conjugates with one of the Lzs, and a CPP conjugated with the other Lz. Using them, we showed that even when both the cargo and the CPP were linked to Lz, the expected interaction between LzK and LzE occurred; that is, LzK/LzE was used as a molecular zipper to indirectly link a functional peptide or protein with CPP. It was also revealed that the formed hybrid could deliver cargo into cells in a CPP-dependent manner. Furthermore, the delivered cargo functioned intracellularly as expected. These features are superior to those of cargo directly linked to CPP [16,19].

Although our attempt to use LzK/LzE was successful, the next important step is to adjust the chain length of the heterodimeric leucine zipper peptides. Especially, we desire to obtain a shorter heterodimeric leucine zipper applicable to this method. At present, the chain length of the peptides required for functioning as a molecular zipper is unknown. The reported length of each of the Lz peptides constituting LzK/LzE is 29 mer. Considering that the maximum peptide length that can be synthesized by the SPPS method is about 50 mer, the functional peptide chain length must be about 20 mer or less. Although this restriction on functional peptide length greatly limits the choice of peptides to be delivered to cells by the Lz hybrid method, successful peptide shortening would help to reduce the time and cost of synthesis. Furthermore, we will be able to have longer chain length functional peptides [26] by this method.

In this study, other heterodimeric leucine zipper peptides with different chain lengths consisting of 1–4 repeating units, (KIAALKE)n (Kn) and (EIAALEK)n (En) (n = 1, 2, 3, and 4), were used [27]. These peptides are also leucine zipper peptides that form specific 1:1 hybrids, similar to Lz, and several research groups have used them for applied biochemical research [11,28,29,30,31,32,33]. We synthesized peptides in which AIP [34] or CPP was linked to Kn or En having various numbers of repeating units and assessed their properties. By evaluating the stability of hybrids formed between AIP-Kn and En-CPP, the efficiency of transducing hybrids into cells, and autophagy induction inside cells, the smallest number of repeating units that can effectively function as a molecular zipper was revealed.

## 2. Materials and Methods

### 2.1. Materials

We purchased 9-Fluorenylmethyloxycarbonyl group (Fmoc)-derivatized amino acids as building blocks for peptides from Watanabe Chemicals (Hiroshima, Japan). Fmoc-derivatized super-acid-labile poly(ethylene)glycol (Fmoc-NH-SAL-PEG) resin, piperidine, O-(1H-benzotriazol-1-yl)-N,N,N′,N′-tetramethyluronium hexafluorophosphate (HBTU) and N-methylmorpholine (NMM) for deprotection/coupling processes for peptide synthesis, and trifluoroacetic acid (TFA) and triisopropylsilane (TIPS) as reagents for the cleavage of peptides on the resin, were also purchased from Watanabe Chemicals. We purchased fluorescent dyes 5(6)-fluorescein (Fam) and 5(6)-tetramethylrhodamine (Tmr) to assess the intracellular delivery of the peptides, from AAT Bioquest (Sunnyvale, CA, USA). N,N′-Dimethylformamide (DMF), diethyl ether, and acetonitrile were purchased from FUJIFILM Wako Pure Chemical Corporation (Osaka, Japan). N,N′-Dimethylsulfoxide (DMSO) was purchased from Nacalai Tesque (Kyoto, Japan). We purchased Dulbecco’s Modified Eagle’s Medium (DMEM), fetal bovine serum (FBS), and Opti-MEM from Thermo Fisher Scientific (Waltham, MA, USA). All reagents were used without further purification.

### 2.2. Antibodies

Anti-actin monoclonal antibody (C4) and anti-LC-3 antibody were purchased from Santa Cruz Biotechnology (Dallas, TX, USA) and Medical & Biological Laboratories (MBL; Nagoya, Japan), respectively. As the secondary antibodies, we used horseradish peroxidase (HRP)-conjugated anti-mouse IgG (Thermo Fisher Scientific) for anti-actin monoclonal antibody (C4), and HRP-conjugated anti-rabbit IgG (Thermo Fisher Scientific) for anti-LC3 antibody. In Western blotting, the primary antibodies were diluted 1000-fold and the secondary antibodies were diluted 3000-fold with Tris-buffered saline with 0.05% Tween 20 (TBS-T).

### 2.3. Peptide Synthesis

**Figure 1:**
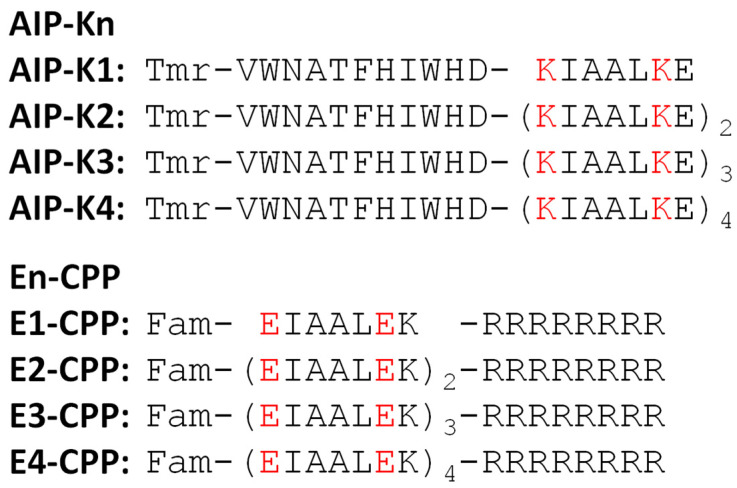
Sequences of eight fluorescently labeled coiled-coil peptides, AIP-Kn and En-CPPs (n = 1–4). The red letters indicate the charged amino acids critical for the formation of a 1:1 hybrid between Kn and En peptides. Tmr and Fam stand for the fluorescent dyes tetramethylrhodamine and fluorescein, respectively.

We synthesized AIP-Kn and En-CPP peptides (Figure 1) on Fmoc-NH-SAL-PEG resin containing 7.2 μmol (1 eq) Fmoc on the resin surface using conventional Fmoc-based SPPS. The Fmoc deprotection was performed using 20% piperidine in DMF for 7 min and then washed with DMF (×3). Deprotection and coupling processes were performed at room temperature without a capping process. For each coupling process, Fmoc-derivatized amino acids or fluorescent dyes (Fam and Tmr; 4 eq), HBTU (3.6 eq), and NMM (11.5 eq) were dissolved in DMF and added to the resin. The reaction was allowed to proceed for 45 min and then washed with DMF (×3). After repeated cycles of deprotection/coupling until the desired length of peptide was produced, we performed global deprotection of protecting groups on the peptide side chain and cleavage of the peptides from the resin by treatment with TFA:TIPS:water [95:2.5:2.5 (*v*/*v*)] for 90 min at room temperature. We precipitated the obtained crude peptides in diethyl ether and washed with ether (×2) until neutral pH was reached. The recovered peptides were dried with air and dissolved in 50:50 (*v*/*v*) acetonitrile/0.1% TFA in water for purification.

We conducted the purification of peptides on a reverse-phase high performance liquid chromatography (RP-HPLC). The RP-HPLC was equipped with two prominence LC pumps (LC-20AT) and a SPD-20A prominence UV/Vis detector (Shimadzu, Kyoto, Japan). We carried out data acquisition and processing with Chromato-PRO (Runtime Instruments, Tokyo, Japan). The preparative column was Cadenza 5CD-C18 column (250 × 20 mm i.d.; Imtakt, Kyoto, Japan). The mobile phase consisted of 0.1% TFA aq. (A solvent) and acetonitrile (B solvent). We used as linear gradient from 5% to 50% of B solvent at the flow rate of 10.0 mL/min (25 °C). The sample injection volume was 4 mL, run time was 30 min and detection was at 230 nm. Final product identification was analyzed with the HPLC system mentioned above. The analytical column was using Cadenza CD-C18 column (75 × 4.6 mm i.d.; Imtakt). We used as linear gradient from 10% to 80% of B solvent at the flow rate of 1.0 mL/min (25 °C). The sample injection volume was 0.1 mL, run time was 20 min and detection was at 230 nm. (see Figures S1–S8 in Supplementary Materials). The purity of all peptides used in this study was 98% or above (See Table S1 in Supplementary Materials). The final product identification was also with performed matrix-assisted laser desorption/ionization-time-of-flight (MALDI-TOF) mass spectrometry (Shimadzu AXIMA Confidence). Observed exact masses were obtained in linear positive mode using a-cyano-4-hydroxycinnamic acid as the matrix (see Figures S9–S16 in Supplementary Materials).

### 2.4. Fluorescence Titration Curves

The fluorescence levels of AIP-Kn/En-CPP (n = 1, 2, 3, and 4) mixtures in PBS (pH 7.0) were measured using a fluorescence spectrometer (FP-8200; JASCO, Tokyo, Japan) and a quartz cell with a length of 1 cm. The concentration of En-CPP was maintained at 50 nM while concentrations of AIP-Kn were varied in order to create Tmr/Fam (AIP-Kn/En-CPP) ratios of 0, 0.2, 0.4, 0.6, 0.8, 1.0, 1.2, 1.4, 1.6, 1.8, and 2.0. Fluorescence intensities were measured at 25 °C using an excitation wavelength of 495 nm to excite the Fam and emission wavelengths from 500 to 700 nm. Fluorescence titration curves were recorded based on fluorescence intensity at 525 nm. Binding parameter values (*K*_d_) were obtained by fitting the titration curves to the following equation [35,36].

(1) $$F=F_{0}-(F_{0}-F_{s})\frac{\left(P_{1}+P_{2}+K_{d}\right)-\sqrt{{\left(P_{1}+P_{2}+K_{d}\right)}^{2}-4P_{1}P_{2}}}{2P_{1}}$$

In this equation, *F* is the relative fluorescence intensity of En-CPP when AIP-Kn is added, *F*_0_ is the fluorescence intensity of the initial concentration of Fam, while *F*_s_ is the fluorescence intensity at saturation obtained from AIP-K4/E4-CPP. This processing was performed because in AIP-K3/E3-CPP, the decrease in the fluorescence intensity of E3-CPP did not sufficiently reach saturation by the addition of AIP-K3. *P*_1_ is the fixed concentration of En-CPP (50 nM), *P*_2_ is the concentration of AIP-Kn, and *K*_d_ is the dissociation constant of AIP-Kn/En-CPP hybrid.

### 2.5. Cell Culture

HeLa cells were cultured in DMEM supplemented with 10% heat-inactivated FBS in a 5% CO_2_ incubator at 37 °C. The HeLa cell line has been stocked and propagated in Dr. Masaaki Miyazawa’s laboratory.

### 2.6. Preparation of the K/E Zippers

AIP-Kn and En-CPP (n = 2, 3, and 4) were dissolved in DMSO at 10 mM each to make a stock peptide solution. The stock solution was diluted to the desired concentration using Opti-MEM. Equal volumes of the diluted AIP-Kn and En-CPP were mixed in a 1.5 mL tube for 1 h with rotation at room temperature to generate hybrids. For single-component controls (AIP-Kn or En-CPP only), equal volumes of the diluted AIP-Kn or En-CPP and Opti-MEM with DMSO, which was added instead of the stock peptide solution, were mixed in a 1.5 mL tube, and the mixture was then treated in a manner similar to that for the hybrid formation. A vehicle control was established using Opti-MEM with DMSO, which was also treated in a manner similar to that for the hybrid formation.

### 2.7. Evaluation of the K/E Hybrid Delivery into Cells

One day before peptide treatment, 1 × 10^5^ cells in 2 mL of HeLa cells were seeded on a glass-bottomed dish (Matsunami Glass, Osaka, Japan) and cultured in a 5% CO_2_ incubator at 37 °C. On the next day, before peptide treatment, the cell supernatant was removed by aspiration, and the cells were washed with 2 mL of PBS once. The possible Kn/En hybrids that were prepared by an equimolar mixture of AIP-Kn and En-CPP or vehicle control were added to the washed cells, and the cells were incubated for 1 h in a 5% CO_2_ incubator at 37 °C. After the cells were washed with 2 mL of PBS, these cells were fixed with 2% paraformaldehyde/PBS for 15 min at room temperature and washed three times with PBS. The reason why we fixed the cells is that all samples must have the same incubation time after the addition of the hybrids or controls. The different incubation times could result in distinct delivery efficiency. After washing the cells, 2 mL of PBS was added and fluorescence signals from the cells were observed using a confocal laser microscope with a Plan Apo 60× oil-immersion DIC H objective (CLMS; C2si Ready; Nikon, Tokyo, Japan). Data were analyzed using NIS-Elements Advanced research software (Nikon).

### 2.8. Analysis of Cellular Autophagic Activity

HeLa cells (3 × 10^4^ cells) were seeded in a well of a 24-well plate. One day later, the cells were washed with 1 mL of PBS once and treated with 500 μL of 10 μM Kn/En hybrids, single-component controls, or vehicle control for 3 h in a CO_2_ incubator at 37 °C. The supernatant of cells was removed and washed with 1 mL of PBS. The washed cells were lysed for 30 min at 4 °C in a buffer that consists of 10 mM Tris-HCl at pH 8.0, 150 mM NaCl, 2 mM EDTA, 0.5% NP40, and cOmplete ULTRA protease inhibitor cocktail (Roche, Mannheim, Germany). The cell lysates were centrifuged at 10,000 rpm for 10 min at 4 °C, after which the cleared lysates were mixed with the SDS-PAGE sample buffer that contained 2 mercaptoethanol as the reducing reagent. The mixture was heated at 100 °C for 5 min on a heat block and subsequently subjected to SDS-PAGE. The cellular proteins in the mixture were separated in the polyacrylamide gel. After electrophoresis, the proteins were transferred to a methanol-activated PVDF membrane (Merck Millipore, Darmstadt, Germany) by a tank transfer system (Bio-Rad, Tokyo, Japan). The membrane on which the proteins were transferred was washed with TBS-T for 30 min and probed with a primary antibody for 2 h at room temperature. After washing the membrane with TBS-T three times, the membrane was next incubated with the HRP-conjugated secondary antibody for 1 h at room temperature. After washing the membrane with TBS-T three times, the Luminata Forte western HRP substrate (Merck Millipore) was added, and the protein band signals were detected with the ImageQuant LAS 4010 system (GE Healthcare, Chicago, IL, USA). The ImageQuant TL software (GE Healthcare) was utilized for quantifying band intensities. The band intensities of LC3-II and actin were used to calculate the ratios of LC3-II/actin in order to evaluate autophagy induction by peptides used in this study.

### 2.9. Cytotoxicity Assay

HeLa cells (1 × 10^4^ cells) were seeded in a well of a 96-well plate. One day later, the cells were washed with PBS once and treated with 100 μL of 10 μM Kn/En hybrids, Kn peptides, En peptides, vehicle control, or Opti-MEM as a medium control for 3 h in a 5% CO_2_ incubator at 37 °C. The supernatant was transferred to a new 96-well plate and centrifuged at 1200 rpm for 5 min to ensure that the cell debris settled at the bottom. The cleared supernatants were used to quantify the cytotoxicity. The CytoTox96 non-radioactive cytotoxicity assay (Promega, Madison, WI, USA) was utilized to evaluate the cytotoxicity by treatment of Kn/En hybrid, Kn peptide, and En peptides. This assay was based on the detection of lactate dehydrogenase (LDH) that retains in the cytoplasm when cells are not damaged but leaks out when cells are damaged.

## 3. Results and Discussion

### 3.1. Chemical Structures of Fluorescently Labeled Hetrodimeric Coiled-Coil Modified with AIP or CPP

We designed an autophagy-inducing peptide (AIP) modified with a KIAALKE unit(s) (Kn) at its C-terminus and a cell-penetrating peptide (CPP) octaarginine [37,38,39] modified with an EIAALEK unit(s) (En) at its N-terminus (Figure 1). These eight AIP-Kn and En-CPP were further labeled with the fluorescent dyes tetramethylrhodamine (Tmr) and fluorescein (Fam) at their N-termini, respectively. Here, the amino acid sequence of AIP is VWNATFHIWHD, an 11 mer peptide that was previously reported by Peraro et al. [34,40,41,42]. The n in Kn and En indicates the number of repeats of those units, where n = 1, 2, 3, and 4. Kn and En have been demonstrated to hybridize in a parallel orientation in several studies [43,44]. We expected that, by placing Kn and En at the N- or C-termini of their respective peptides, they would not interfere with each other’s functions.

### 3.2. Comparison of Binding Constant of the AIP-Kn/En-CPP Hybrids

**Figure 2:**
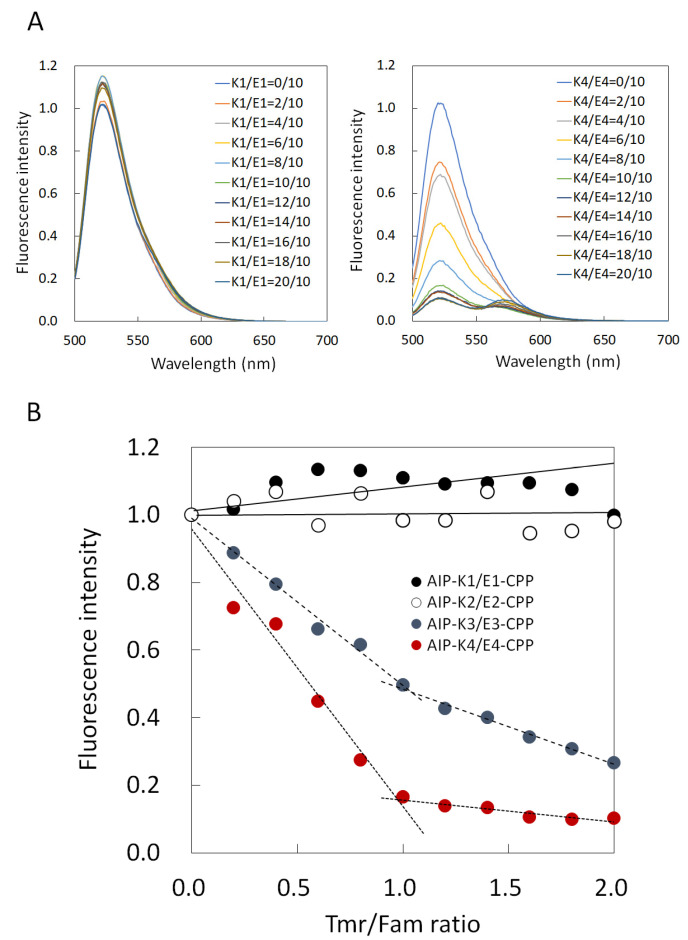
(**A**) Fluorescence spectra of E1-CPP (left panel) and E4-CPP (right panel) with various concentrations of AIP-K1 and AIP-K4, respectively. Fluorescence spectra of AIP-K2/E2-CPP and AIP-K3/E3-CPP are shown in Figures S17 and S18 in the Supplementary Materials. (**B**) Titration curves of fluorescence intensity at 525 nm for Tmr/Fam (AIP-Kn/En-CPP) ratios (n = 1, 2, 3, and 4 represented by black filled squares, black open squares, blue filled circles, and red filled circles, respectively).

First, we obtained the fluorescence spectra of mixtures of four types of AIP-Kn and En-CPP (n = 1, 2, 3, and 4) in an aqueous solution to assess the stability of these hybrids. Figure 2A shows two representative spectra of them (AIP-K1/E1-CPP and AIP-K4/E4-CPP). The left panel shows the results of AIP-K1/E1-CPP. When Tmr in AIP-K1 was gradually added up to 2 equivalents while keeping the concentration of E1-CPP constant, Fam-derived fluorescence intensity at 525 nm in E1-CPP hardly changed. On the other hand, in the case of AIP-K4/E4-CPP, the addition of Tmr in AIP-K4 significantly reduced the Fam-derived fluorescence intensity in E4-CPP (right panel in Figure 2A). In the right panel of Figure 2A, the Tmr-derived fluorescence intensity does not appear to increase when the AIP-K4/E4-CPP (Tmr/Fam) ratio is less than 1, while the fluorescence intensity appears to increase when the Tmr/Fam ratio is greater than 1. This result may be attributed to the fact that Tmr emits little fluorescence due to FRET under this condition, whereas Tmr emits a little fluorescence with excitation at 495 nm. That is, when the Tmr/Fam ratio is 1 or less, the decrease in Fam-derived fluorescence by FRET overlaps with the increase in Tmr-derived fluorescence at 495 nm excitation, and apparently no increase in fluorescence near 570 nm is observed. On the other hand, when the Tmr/Fam ratio is 1 or more, a monotonic increase in fluorescence around 570 nm due to the addition of the Tmr-labeled peptide is observed.

Based on these results, we also plotted the fluorescence intensity at 525 nm against the ratio of Tmr/Fam (AIP-Kn/En-CPP) to clarify the change of fluorescence of Fam in En-CPP with the addition of Tmr in AIP-Kn (Figure 2B). The results revealed that both AIP-K1/E1-CPP and AIP-K2/E2-CPP showed marginal change in Fam fluorescence intensity within this measurement range. On the other hand, in AIP-K3/E3-CPP, when AIP-K3 was gradually added, the fluorescence intensity rapidly decreased to 1 equivalent, above which it continued to decrease, albeit more gradually. In the case of AIP-K4/E4-CPP, the fluorescence intensity decreased more rapidly to 1 equivalent, above which it continued to decrease but very gradually.

These results indicate that Fam approaches Tmr due to the hybrid formation of the E3/K3 pair and E4/K4 pair, resulting in a decrease in Fam fluorescence following Förster/fluorescence resonance energy transfer (FRET) from Fam to Tmr. On the other hand, the findings also indicate that FRET did not occur for the E1/K1 pair or E2/K2 pair. That is, AIP-K3/E3-CPP and AIP-K4/E4-CPP form hybrids under these measurement conditions, while no hybridization proceeded for AIP-K1/E1-CPP and AIP-K2/E2-CPP. In addition, the results of titration curves indicated that AIP-K3/E3-CPP and AIP-K4/E4-CPP form hybrids at a ratio of 1:1. These results clarify that the stability of hybrids between En and Kn depends on the number of repeats (n) of EIAALEK and KIAALKE. Although 1:1 hybridization of E3/K3 and E4/K4 peptides has previously been reported [11,28,29,30,31,32,33], this assessment revealed that, even if AIP and CPP peptides were modified with E3/K3 and E4/K4 peptides, the hybridization was not inhibited.

Next, on the basis of the obtained titration curves, we estimated the dissociation constants (*K*_d_) of AIP-K3/E3-CPP and AIP-K4/E4-CPP hybrids from the fluorescence titration curves using equation (1), and found them to be 2 × 10^−8^ M and 2 × 10^−9^ M, respectively. We previously reported that a hybrid of AIP-LzK with LzE-CPP involving hydrophobic and electrostatic interactions similar to those in this K/E zipper was successfully delivered into cells [16,19]. The *K*_d_ value of the Lz motif-based hybrid without conjugation of AIP or CPP was 4 × 10^−9^ M. The interaction between the 28 mer peptides of AIP-K4/E4-CPP had a stability similar to that of the interaction of Lz motifs.

### 3.3. Intracellular Delivery of AIP-Kn/En-CPP Hybrids

**Figure 3:**
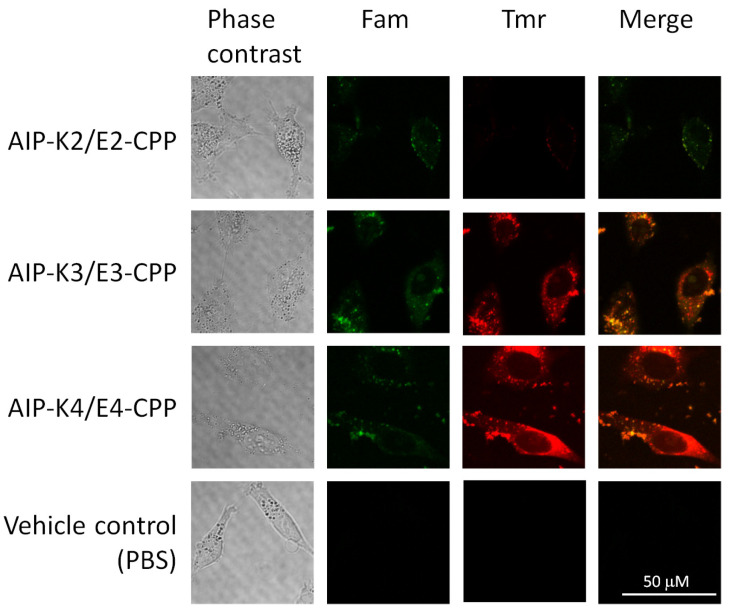
Confocal microscopy images of HeLa cells treated with Kn-/En-based 1:1 mixture. The cells were treated with 10 μM of the respective hybrids, AIP-K2/E2-CPP, AIP-K3/E3-CPP, and AIP-K4/E4-CPP, for 1 h. Fam and Tmr fluorescence signals were separately detected as green and red signals, respectively. Merged images are also presented on the right (yellow signal).

To examine whether the AIP-Kn/En-CPP hybrids are delivered into cells, we observed HeLa cells incubated with an equimolar mixture of AIP-Kn and En-CPP (n = 2, 3, and 4) by confocal laser scanning microscopy (CLMS). We did not use AIP-K1 and E1-CPP since these seemed not to form a hybrid in the above experiment (Figure 2). As shown in Figure 3, Fam fluorescence and no significant Tmr fluorescence signal were observed in cells treated with AIP-K2/E2-CPP, indicating that AIP-K2 does hardly form a hybrid with E2-CPP, and E2-CPP alone translocates across the plasma membrane. In contrast, both Fam and Tmr signals were clearly observed in cells treated with the AIP-K3/E3-CPP and AIP-K4/E4-CPP hybrids. These results indicate that AIP-K3 and AIP-K4 were delivered into the cells due to corresponding CPP hybridization. These results correlate well with the results of fluorescence titration curves (Figure 2).

### 3.4. Evaluation of Autophagy Induction by the Hybrid of AIP-Kn and En-CPP

**Figure 4:**
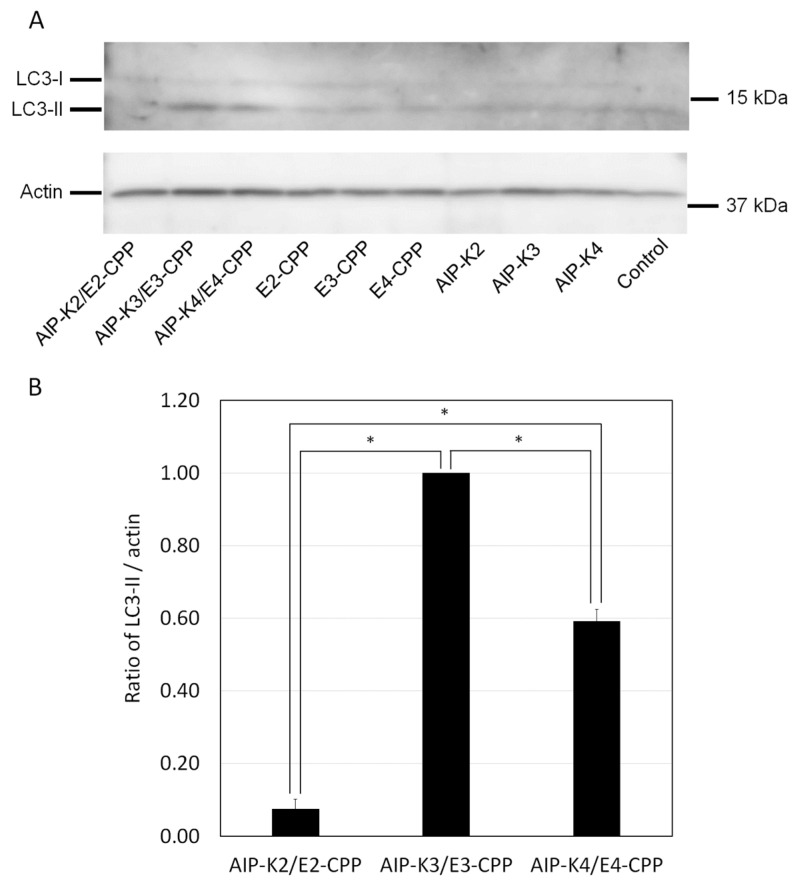
(**A**) Autophagy induction after treatment by the AIP-Kn/En-CPP hybrid, AIP-Kn, and En-CPP (n = 2, 3, and 4). Cells were treated with 10 μM of the indicated hybrids or peptides. Molecular weights of standards are shown on the right side of each membrane. (**B**) Level of the autophagy marker LC3-II in cells. The band intensity of LC3-II and actin in immunoblotting was quantified and the LC3-II/actin ratio was calculated. Actin was used for normalizing the loading amount. The LC3-II/actin ratio for AIP-K3/E3-CPP was set to 1.00. The graph presents the mean with standard error from three independent experiments. * *p* < 0.0001; *p*-values were calculated using one-way ANOVA with Bonferroni’s multiple comparison tests.

Upon successful delivery of AIP-Kn by En-CPP (n = 3 and 4) via K/E hybridization into cells, these peptides should induce autophagy through the well-known AIP activity [18]. To confirm this, we treated cells with Kn/En hybrids and examined autophagy induction by determining the level of the autophagy marker protein LC3-II. When autophagy is induced, LC3-I, a non-lipid form of LC3, is converted into LC3-II via modification with phosphatidylethanolamine. These LC3s are distinguishable on SDS-PAGE and can be detected by immunoblotting using an anti-LC3 antibody. LC3-II was induced in the presence of AIP-K3/E3-CPP and AIP-K4/E4-CPP hybrids, but not when En-CPP or AIP-Kn (n = 2, 3, and 4) alone or an AIP-K2/E2-CPP mixture was present (Figure 4A). We compared the autophagy induction of AIP-Kn/En-CPP using the LC3-II/actin ratio obtained from the immunoblotting and found that AIP-K2/E2-CPP and AIP-K4/E4-CPP were 0.08 and 0.59, respectively, when the LC3-II/actin ratio of AIP-K3/E3-CPP was set to 1.00 (Figure 4B). These values are about one-tenth and one-half those for AIP-K3/E3-CPP, respectively, indicating that AIP-K3/E3-CPP has the highest autophagy-inducing activity. We also subjected these peptides to the LDH assay and found that none of the samples showed cytotoxicity (Figure S19).

These results indicate that the higher hybrid stability of Kn/En does not reflect its higher autophagy-inducing activity. In other words, the low intracellular hybrid stability of Kn/En may promote the separation of AIP and CPP inside cells, implying that AIP activity is elicited by avoiding the adverse effects of CPP. Thus, these results indicate that the Kn/En hybrid has an optimal chain length. While it is important for the repeat number n to be sufficiently high for delivery into cells, it is also important for n to be sufficiently low for efficient bioactivity inside cells after delivery.

## 4. Conclusions

Previous work showed that the heterodimeric interaction between two types of leucine zipper (Lz) sequence can produce a molecular zipper to indirectly link a functional peptide or protein to a CPP for their intracellular delivery. However, in the intracellular delivery system, the number of repeat units required for functioning as a molecular zipper remains entirely unknown. In the present study, we used Kn and En with distinct repeat units as alternative heterodimeric leucine zippers because the sequence of the repeat units in the Lz are ambiguous.

We synthesized Kn-AIP containing Tmr and En-CPP containing Fam. Upon subjecting them to fluorescence spectroscopy analysis, it was shown that the Kn/En heterodimer formed when the Kn/En repeat units numbered 3 and 4. On the other hand, when the repeat number was 1 or 2, the K/E zipper did not form. Reflecting this result, successful delivery into cells was achieved for the AIP-K3/E3-CPP and AIP-K4/E4-CPP zippers, which induced autophagy, while this did not occur for K2-AIP/E2-CPP. AIP-K3/E3-CPP and AIP-K4/E4-CPP introduced into cells showed the expected induction of autophagy without cytotoxicity, but the activity of AIP-K3/E3-CPP was found to be higher than that of AIP-K4/E4-CPP.

These results concluded that: The Kn/En zipper increases the hybrid stability as the number of n increases, resulting in improved the intracellular delivery of AIP by connection with CPP via the Kn/En zipper. On the other hand, the Kn/En zipper decreases the hybrid stability as the number of n decreases, resulting in the enhanced intracellular activity of AIP by dissociation from CPP. This enhancement is likely due to the avoidance of AIP from non-specific binding to intracellular negatively charged RNAs and proteins by CPPs. The above exquisite balance affects the intracellular activity of AIP, and AIP-K3/E3-CPP ultimately resulted in the most potent autophagy induction in the present study.

Our finding on the appropriate length of the K/E zipper saves time and reduces the cost associated with synthesis, achieves higher bioactivity of functional peptides, and allows for us to utilize longer functional peptides.

## Data Availability

Not applicable.

## Supplementary Materials

The following supporting information can be downloaded at: https://www.mdpi.com/article/10.3390/pharmaceutics15041048/s1, Figures S1–S8: MALDI-TOF Mass spectra of peptides; Figures S9–S16: RP-HPLC charts of peptides; Figures S17 and S18: Fluorescence spectra of AIP-K2/E2-CPP and AIP-K3/E3-CPP; Figure S19: Cytotoxicity assay of peptides; Table S1: Synthesized peptides used in this study: Nomenclature and structure.
