# Supplementary material for: Adjusting Heterodimeric Coiled-Coils (K/E Zipper) to Connect Autophagy-Inducing Peptide with Cell-Penetrating Peptide
Source: Pharmaceutics. 2023 Mar 24;15(4):1048. doi: 10.3390/pharmaceutics15041048 (PMC10141234; doi:10.3390/pharmaceutics15041048)
Supplement: Supplementary file 1 [file pharmaceutics-15-01048-s001.zip › pharmaceutics-2244884-supplementary.pdf]

Supporting Information

Adjusting heterodimeric coiled-coils (K/E zipper) to connect autophagy-inducing peptide with cell-penetrating peptide

Yoshiyuki Hakata [1,2,†] , Kazuma Yamashita [3,†] , Sonoko Hashimoto [3] , Takashi Ohtsuki [4] , Masaaki Miyazawa [1] and Mizuki Kitamatsu [3,*]

1 *Department of Immunology, Faculty of Medicine, Kindai University, 377-2 Ohno-Higashi, Osaka-Sayama, Osaka 589-8511, Japan*
2 *Department of Arts and Sciences, Faculty of Medicine, Kindai University, 377-2 Ohno-Higashi, Osa-ka-Sayama, Osaka 589-8511, Japan*
3 *Department of Applied Chemistry, Faculty of Science and Engineering, Kindai University, 3-4-1 Kowakae, Higashi-Osaka, Osaka 577-8502, Japan*
4 *Department of Interdisciplinary Science and Engineering in Health Systems, Okayama University, 3-1-1 Tsu-shimanaka, Kita-ku, Okayama 700-8530, Japan*
* *Correspondence: kitamatu@apch.kindai.ac.jp*

† *These authors contributed equally.*

Table S1. Synthesized peptides used in this study: Nomenclature and structure[a]

Peptide	Sequence	Exact mass		Purity
		Calculated	Observed	(%)
**AIP-K1**	Tmr-VWNATFHIWHD- KIAALKE	2591.30	2591.27	98
**AIP-K2**	Tmr-VWNATFHIWHD-$(KIAALKE)_2$	3344.78	3343.72	98
**AIP-K3**	Tmr-VWNATFHIWHD-$(KIAALKE)_3$	4098.25	4099.07	98
**AIP-K4**	Tmr-VWNATFHIWHD-$(KIAALKE)_4$	4851.73	4850.63	98
**E1-CPP**	Fam- EIAALEK -RRRRRRRR	2379.31	2379.31	98
**E2-CPP**	Fam-$(EIAALEK)_2$-RRRRRRRR	3133.73	3135.42	98
**E3-CPP**	Fam-$(EIAALEK)_3$-RRRRRRRR	3887.17	3889.92	98
**E4-CPP**	Fam-$(EIAALEK)_4$-RRRRRRRR	4641.59	4642.71	98

[a] The calculated masses and the masses observed by MALDI-TOF mass spectrometry and the purity found by RP-HPLC are listed for each of the peptides.

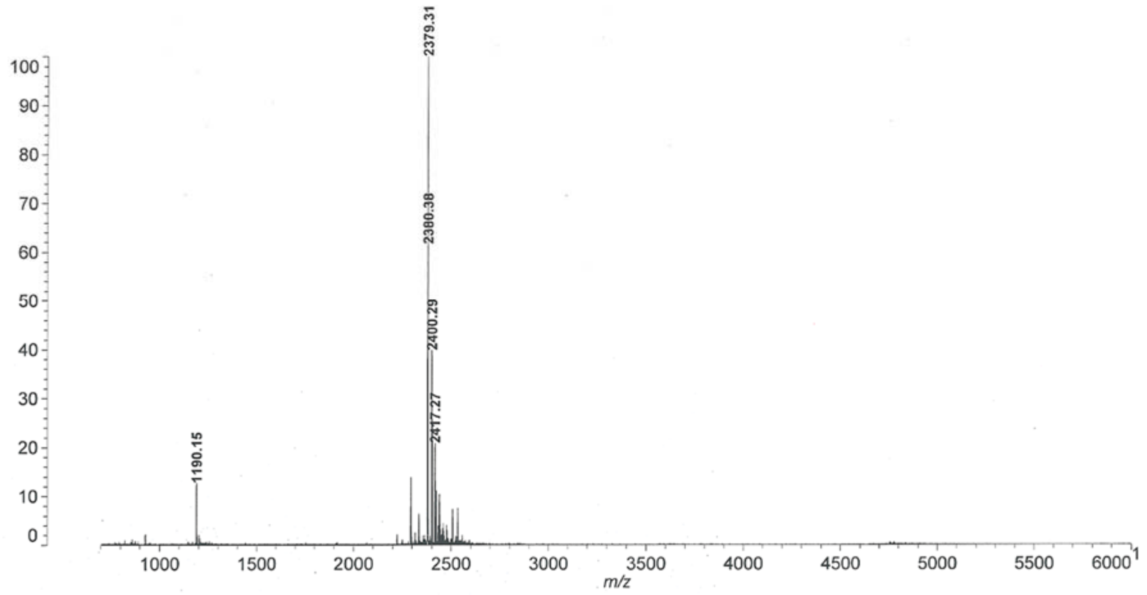

Figure S1. MALDI-Tof Mass spectrum of **E1-CPP**. An α-CHCA was used as a matrix. calcd. $[M+H]^+$ = 2379.31 and obsd. $[M+H]^+$ = 2379.31.

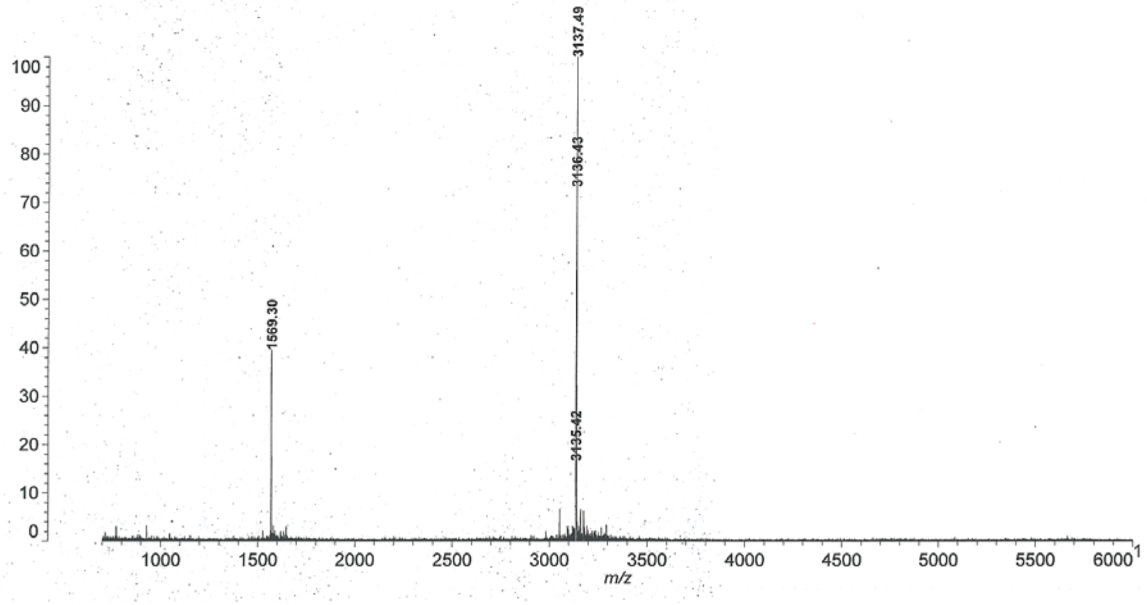

Figure S2. MALDI-Tof Mass spectrum of **E2-CPP**. An α-CHCA was used as a matrix. calcd. $[M+H]^+$ = 3133.73 and obsd. $[M+H]^+$ = 3135.42.

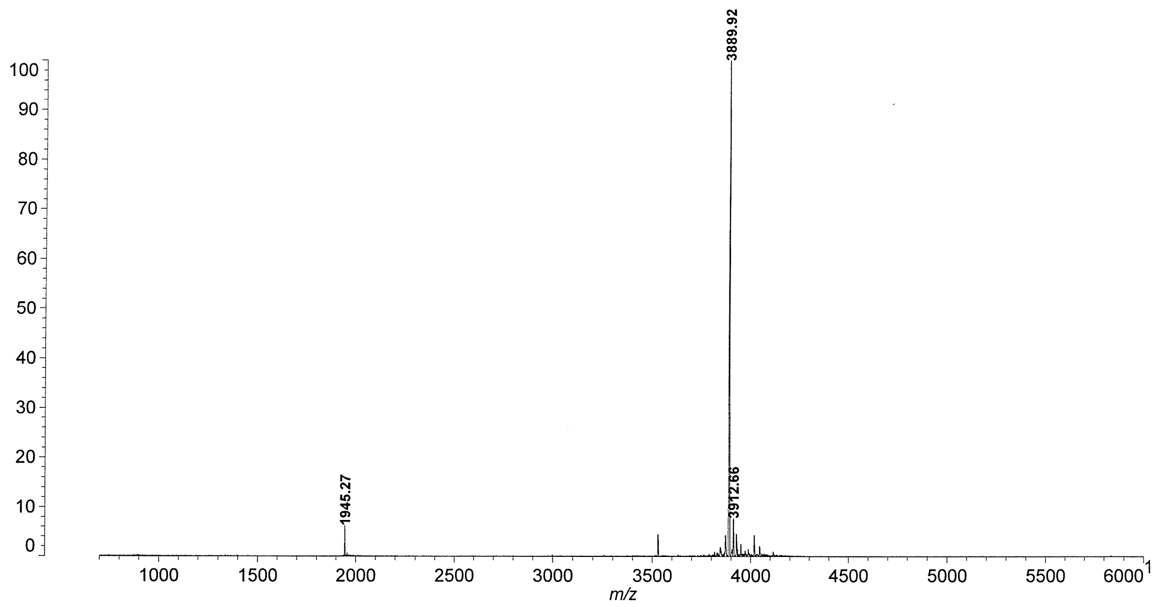

Figure S3. MALDI-Tof Mass spectrum of **E3-CPP**. An α-CHCA was used as a matrix. calcd. $[M+H]^+$ = 3887.17 and obsd. $[M+H]^+$ = 3889.92.

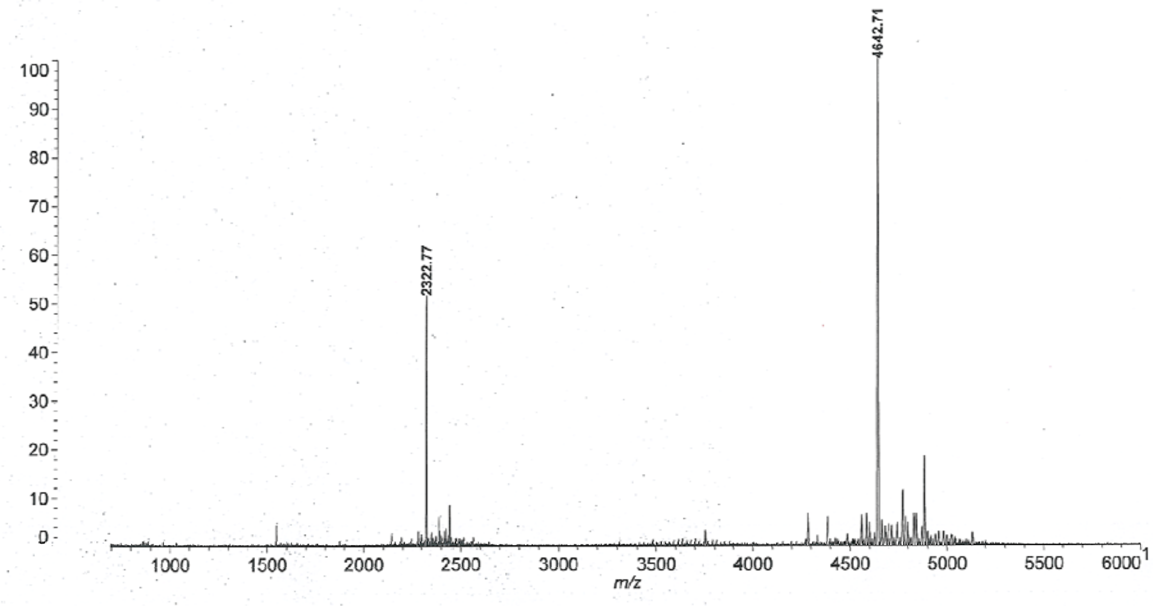

Figure S4. MALDI-Tof Mass spectrum of **E4-CPP**. An α-CHCA was used as a matrix. calcd. $[M+H]^+$ = 4641.59 and obsd. $[M+H]^+$ = 4642.71.

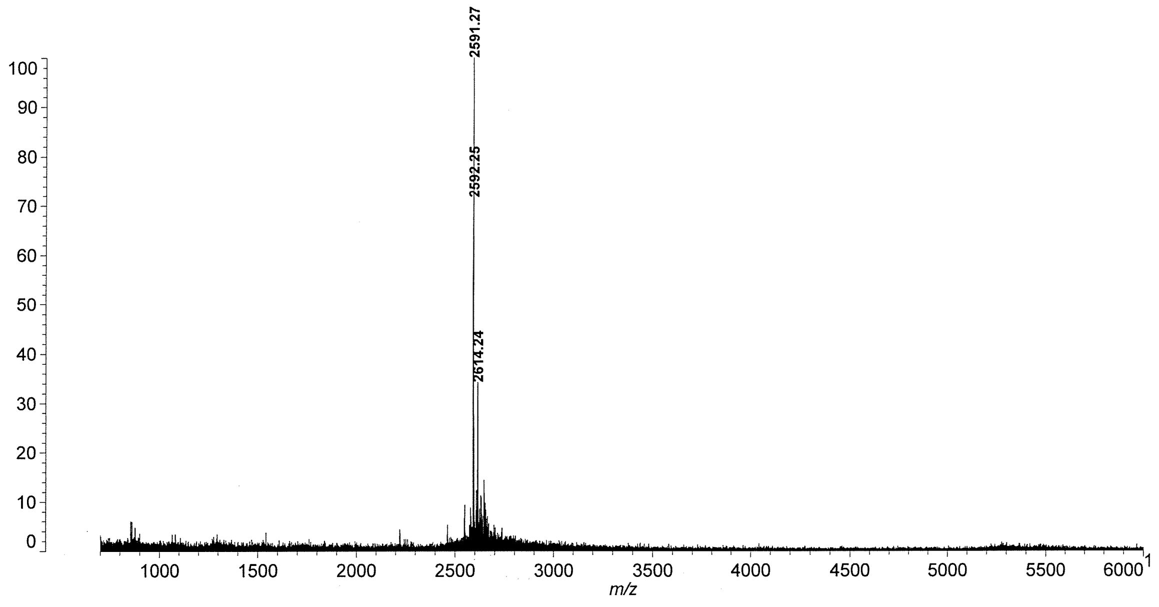

Figure S5. MALDI-Tof Mass spectrum of **AIP-K1**. An α-CHCA was used as a matrix. calcd. $[M+H]^+$ = 2591.30 and obsd. $[M+H]^+$ = 2591.27.

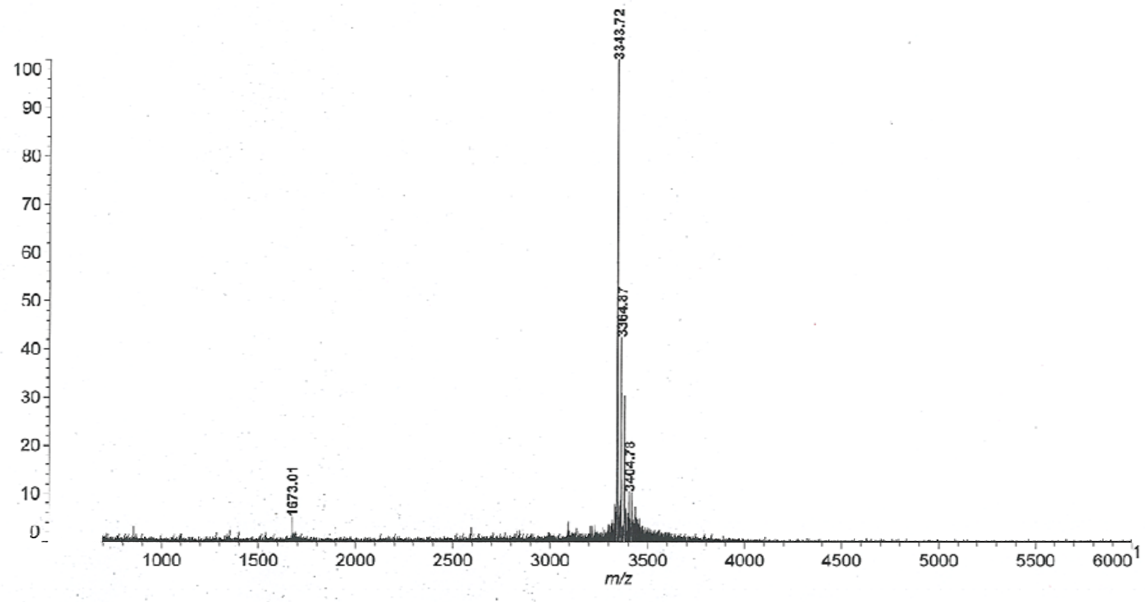

Figure S6. MALDI-Tof Mass spectrum of **AIP-K2**. An α-CHCA was used as a matrix. calcd. $[M+H]^+$ = 3344.78 and obsd. $[M+H]^+$ = 3343.72.

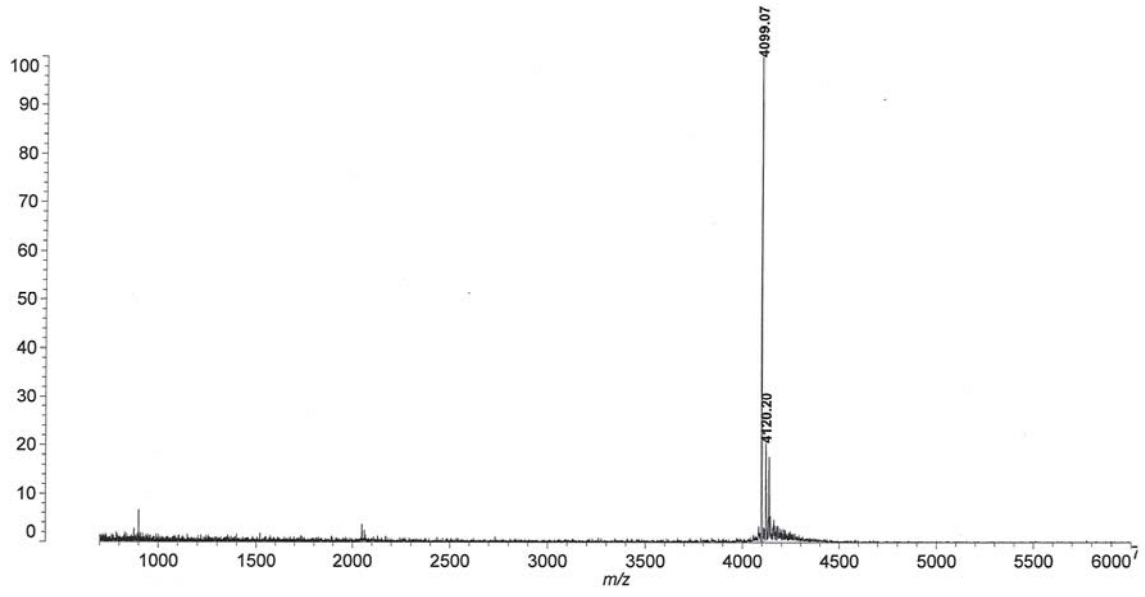

Figure S7. MALDI-Tof Mass spectrum of **AIP-K3**. An α-CHCA was used as a matrix. calcd. $[M+H]^+$ = 4098.25 and obsd. $[M+H]^+$ = 4099.07.

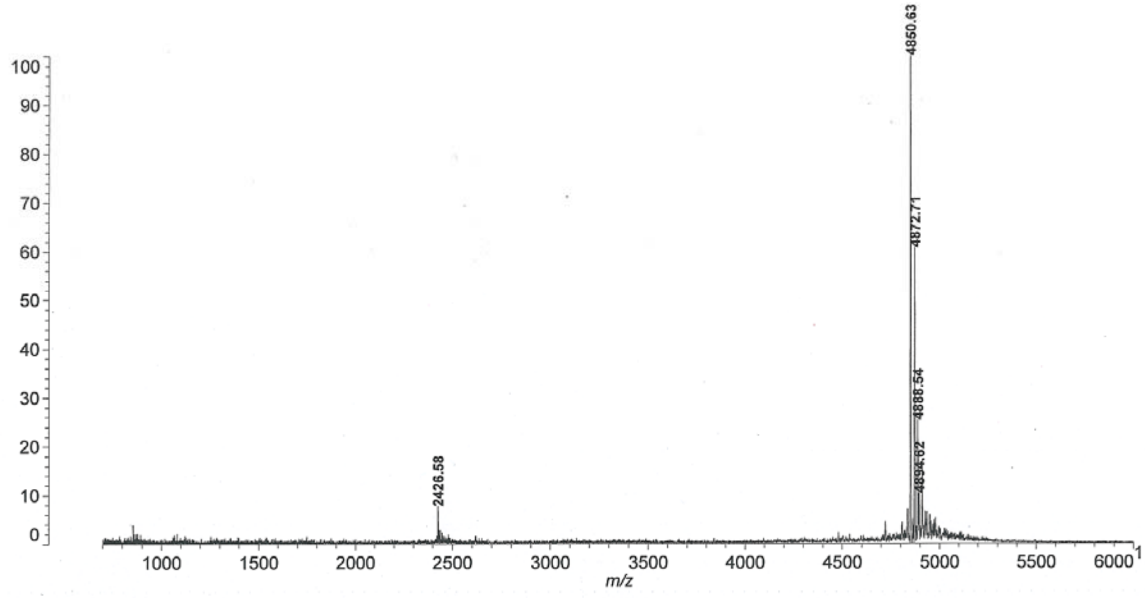

Figure S8. MALDI-Tof Mass spectrum of **AIP-K4**. An α-CHCA was used as a matrix. calcd. $[M+H]^+$ = 4851.73 and obsd. $[M+H]^+$ = 4850.63.

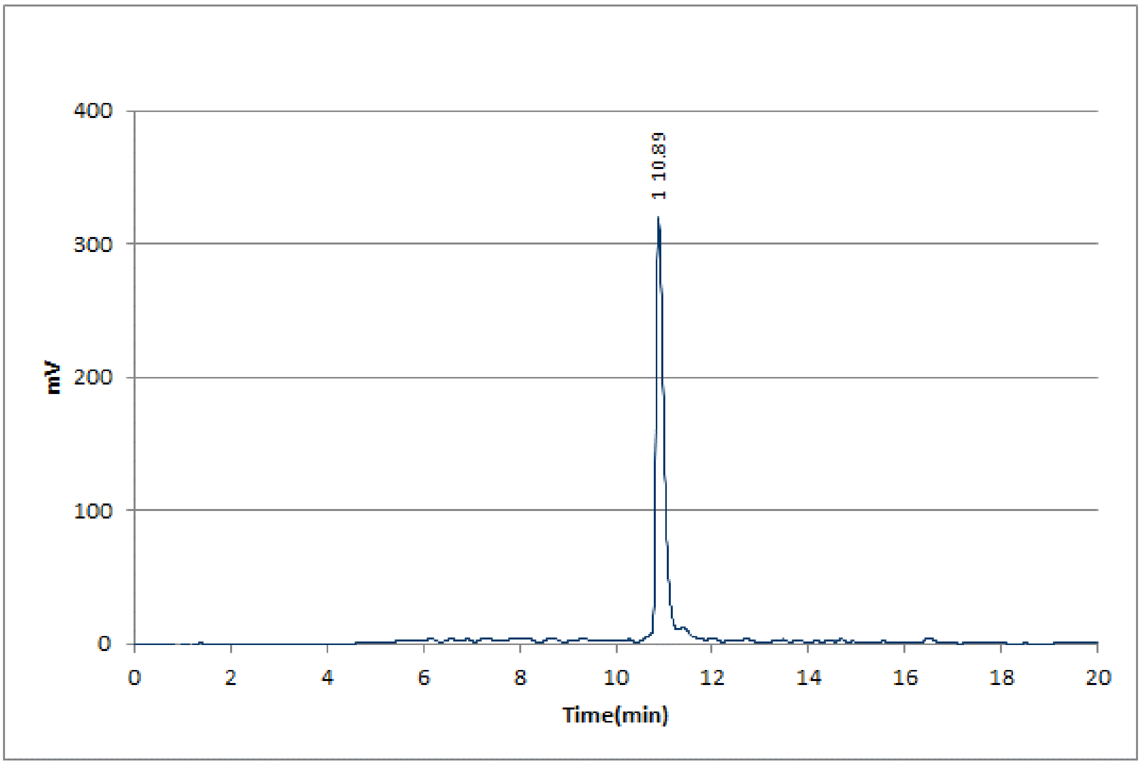

Figure S9. RP-HPLC chart of **E1-CPP** on C18 column. Buffer A. 0.1% TFA in water; buffer B, acetonitrile and monitoring at 230 nm with a gradient of 10-80% for 20 min.

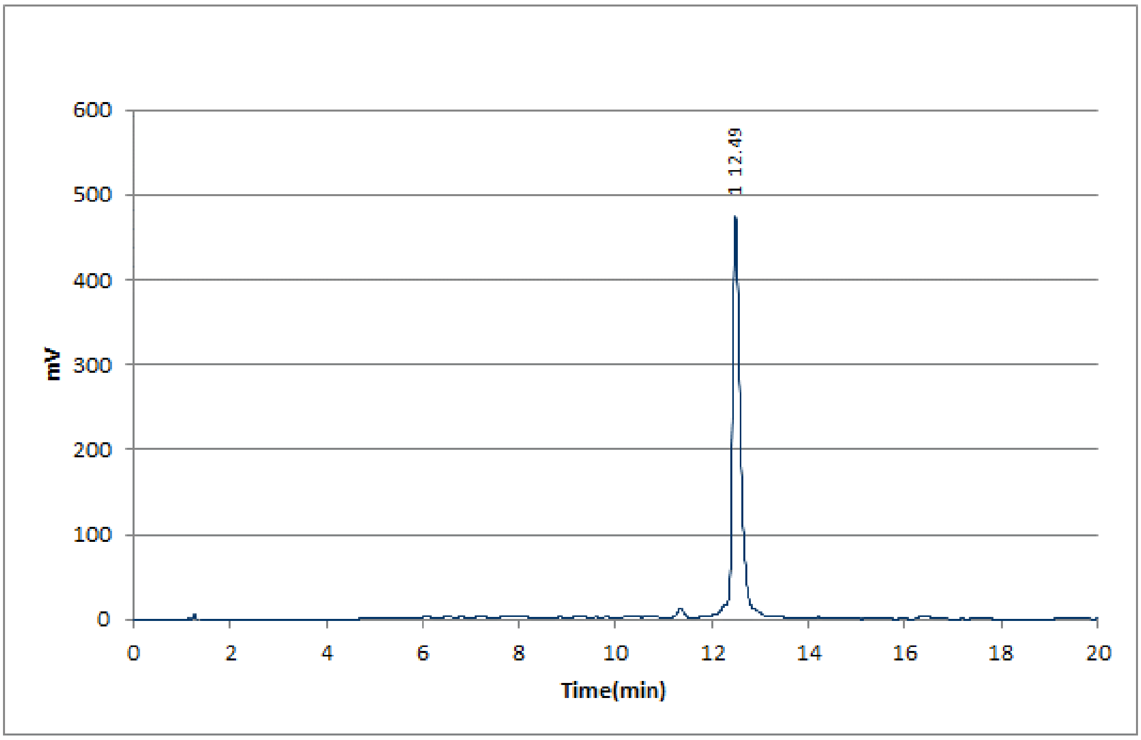

Figure S10. RP-HPLC chart of **E2-CPP** on C18 column. Buffer A. 0.1% TFA in water; buffer B, acetonitrile and monitoring at 230 nm with a gradient of 10-80% for 20 min.

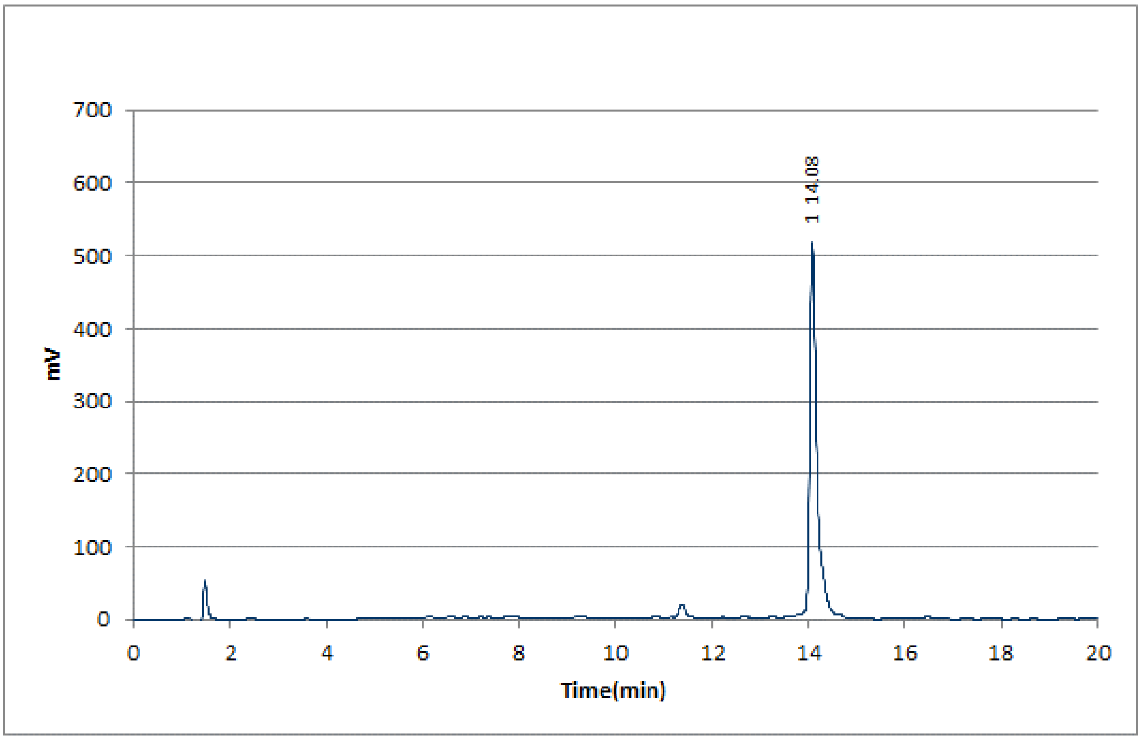

Figure S11. RP-HPLC chart of **E3-CPP** on C18 column. Buffer A. 0.1% TFA in water; buffer B, acetonitrile and monitoring at 230 nm with a gradient of 10-80% for 20 min.

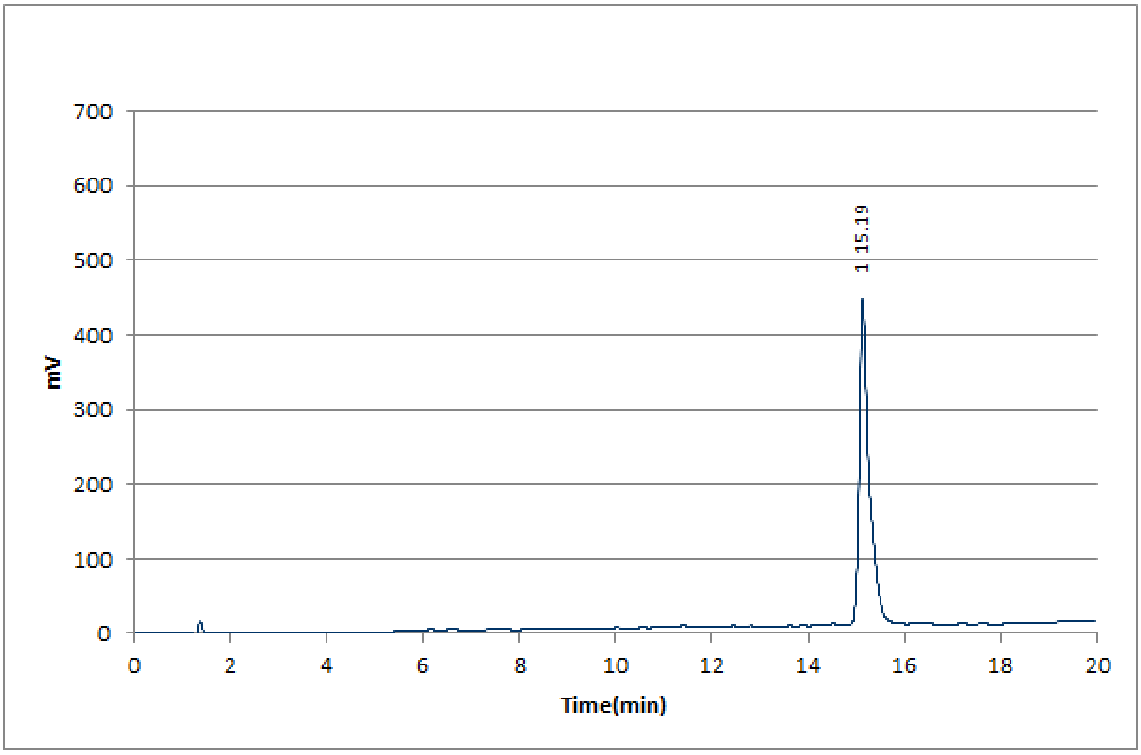

Figure S12. RP-HPLC chart of **E4-CPP** on C18 column. Buffer A. 0.1% TFA in water; buffer B, acetonitrile and monitoring at 230 nm with a gradient of 10-80% for 20 min.

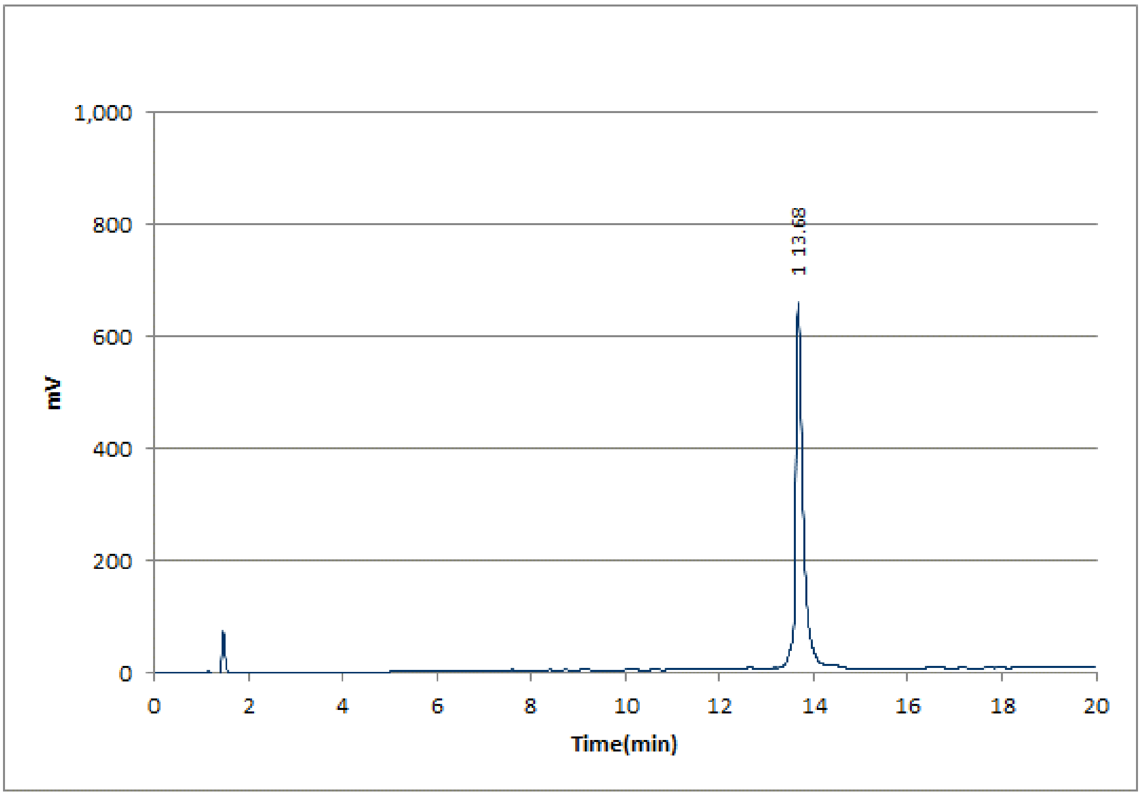

Figure S13. RP-HPLC chart of **AIP-K1** on C18 column. Buffer A. 0.1% TFA in water; buffer B, acetonitrile and monitoring at 230 nm with a gradient of 10-80% for 20 min.

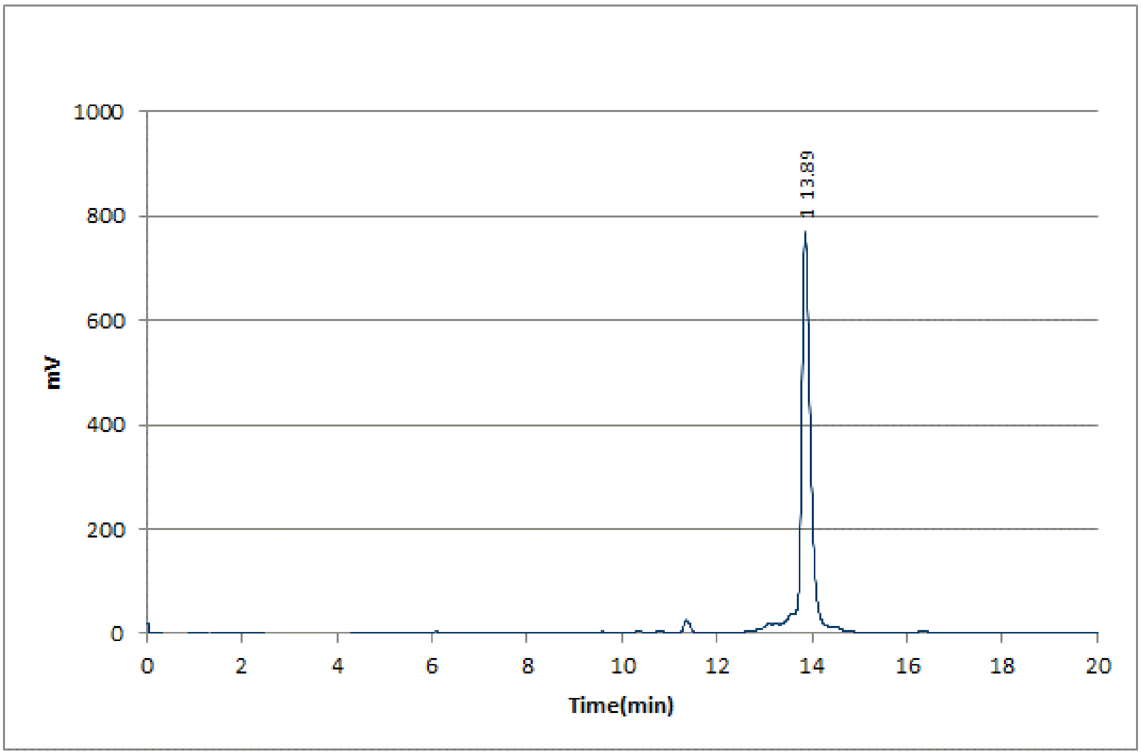

Figure S14. RP-HPLC chart of **AIP-K2** on C18 column. Buffer A. 0.1% TFA in water; buffer B, acetonitrile and monitoring at 230 nm with a gradient of 10-80% for 20 min.

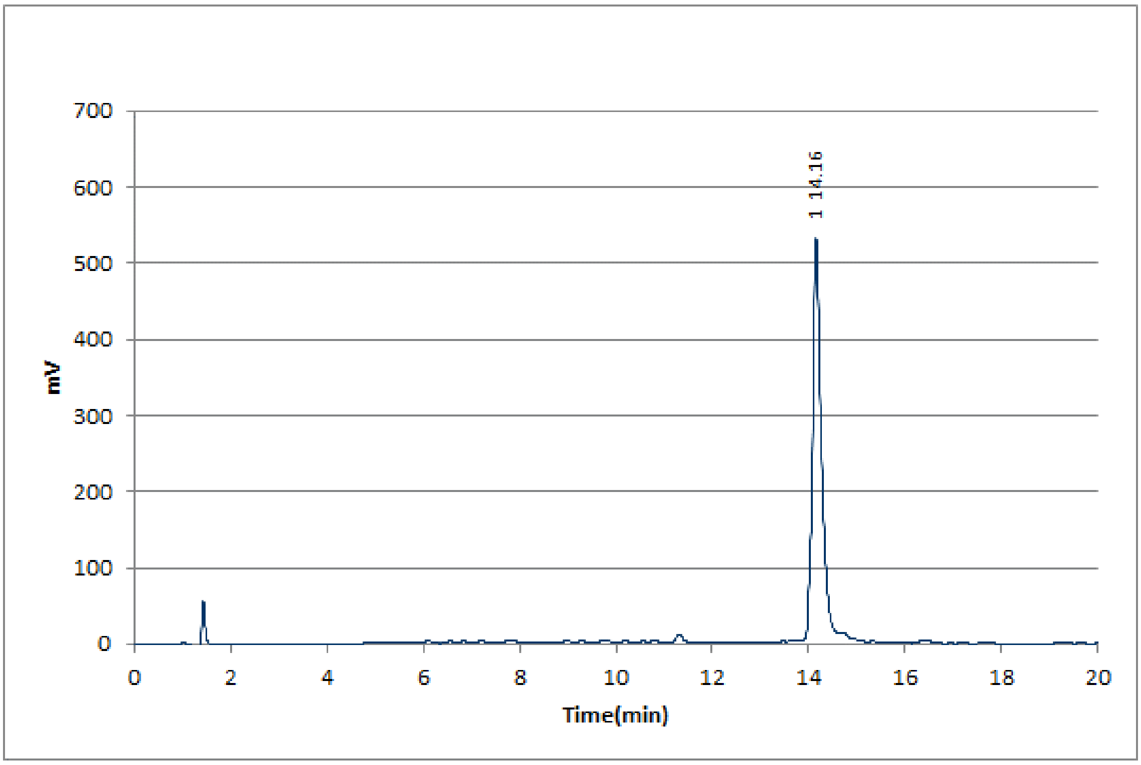

Figure S15. RP-HPLC chart of **AIP-K3** on C18 column. Buffer A. 0.1% TFA in water; buffer B, acetonitrile and monitoring at 230 nm with a gradient of 10-80% for 20 min.

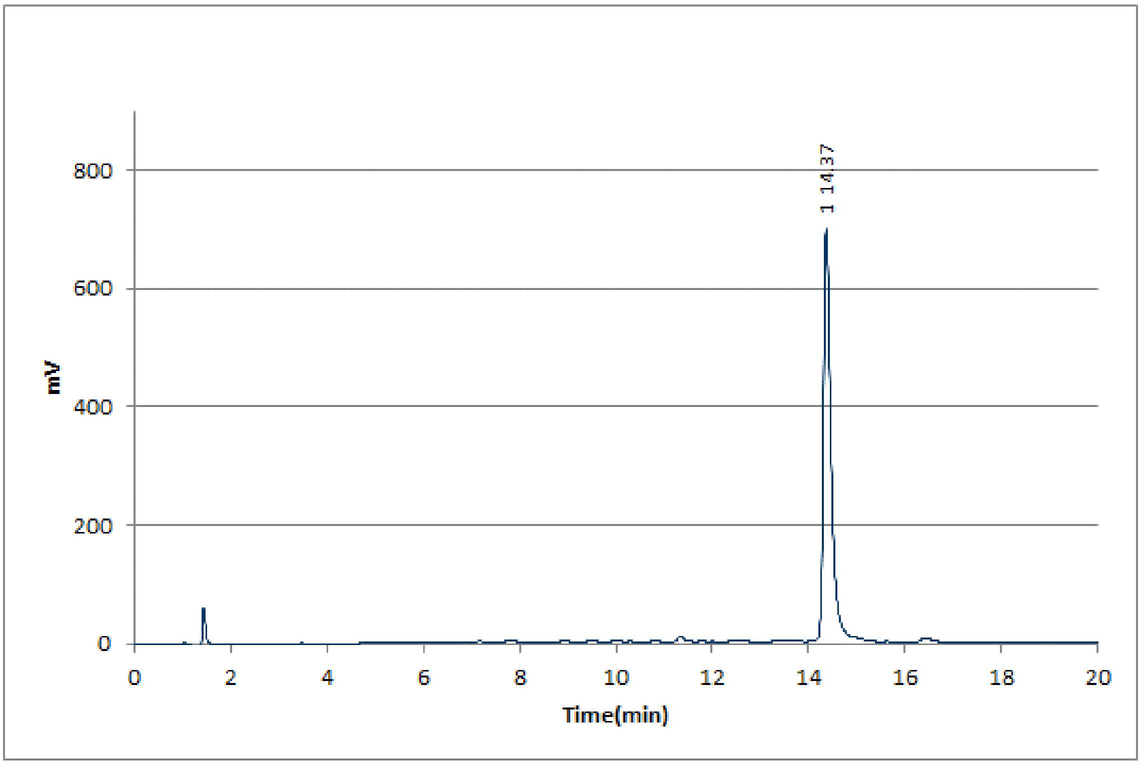

Figure S16. RP-HPLC chart of **AIP-K4** on C18 column. Buffer A. 0.1% TFA in water; buffer B, acetonitrile and monitoring at 230 nm with a gradient of 10-80% for 20 min.

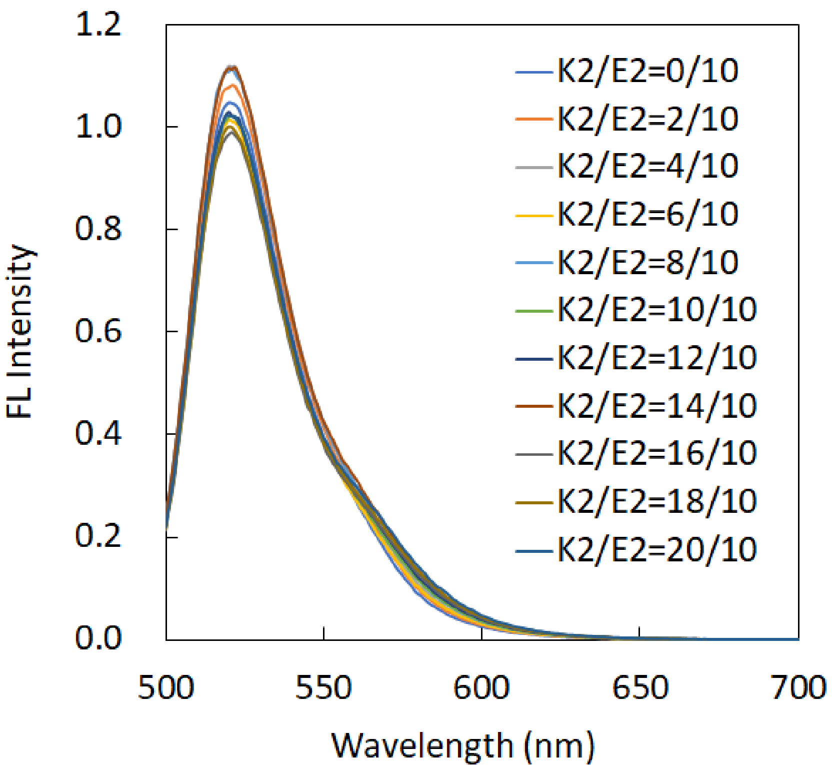

Figure S17. Fluorescence spectra of 50 nM **E2-CPP** with various concentrations of **AIP-K2** in aqueous solution.

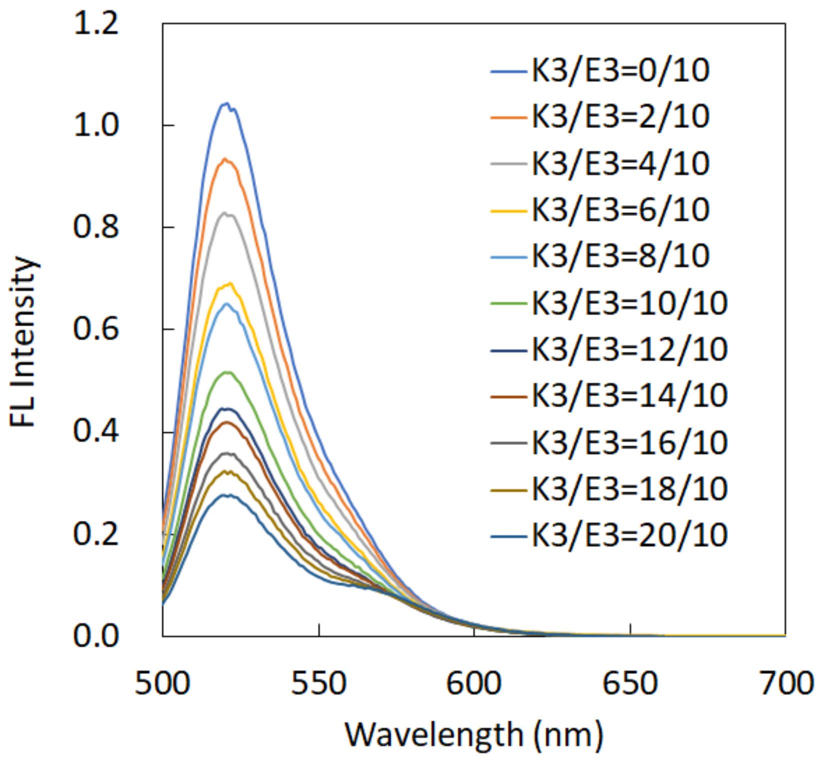

Figure S18. Fluorescence spectra of 50 nM **E3-CPP** with various concentrations of **AIP-K3** in aqueous solution.

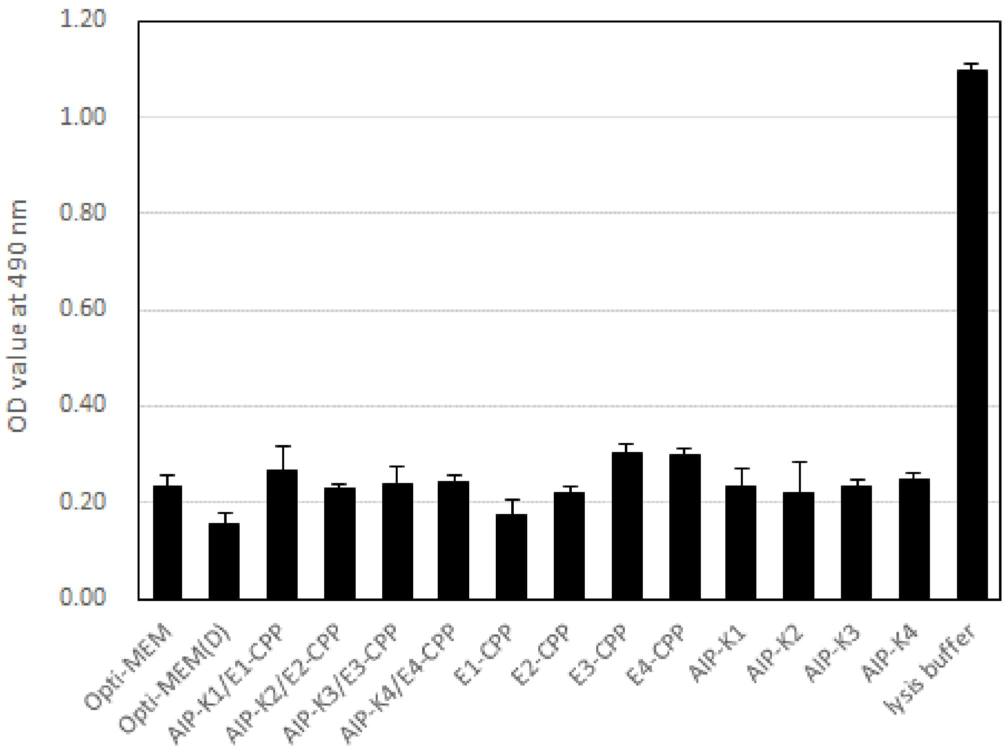

Figure S19. Cytotoxicity of **Kn** peptides, **En** peptides, and **Kn**/**En** hybrids. HeLa cells were treated with 10 μM of the indicated **En**, **Kn** peptides, or **Kn**/**En** hybrids. The cytotoxicity was evaluated with measurement of lactate dehydrogenase in the culture supernatant, which is a stable cytosolic protein and released from cells into the supernatant upon cell lysis. The data represent means with standard errors from three independent experiments.
